# The Association between Hormone Replacement Therapy and Gastroparesis in Post-Menopausal Women: A Worldwide Database Analysis

**DOI:** 10.3390/jpm14030275

**Published:** 2024-02-29

**Authors:** Jacqueline Khalil, Hannah Hill, David Kaelber, Gengqing Song

**Affiliations:** 1 Department of Internal Medicine, MetroHealth Medical Center, Case Western Reserve University, Cleveland, OH 44109, USA; 2 Population Health and Equity Research Institute, MetroHealth Medical Center, Case Western Reserve University, Cleveland, OH 44109, USA; hhill1@metrohealth.org; 3 Center for Clinical Informatics, Research and Education, MetroHealth Medical Center, Department of Internal Medicine, Pediatrics, and Population and Quantitative Health Sciences, Case Western Reserve University, Cleveland, OH 44109, USA; dkaelber@metrohealth.org; 4 Department of Gastroenterology and Hepatology, MetroHealth Medical Center, Case Western Reserve University, Cleveland, OH 44109, USA

**Keywords:** gastroparesis, hormone replacement therapy, post-menopausal women

## Abstract

Female sex hormones have been hypothesized to influence the higher prevalence of gastroparesis in females. This study investigated the effects of hormone replacement therapy (HRT) on gastroparesis and its related symptoms, medication use, and diagnostic testing in post-menopausal women. Utilizing the TriNetX platform, we conducted a population-based cohort study involving post-menopausal women aged 50 or older, with and without HRT. One-to-one propensity score matching was performed to adjust for age, race, ethnicity, diabetes, body mass index (BMI), and hemoglobin A1c. The exclusion criteria included functional dyspepsia, cyclic vomiting syndrome, and surgical procedures. After applying the exclusion criteria, we identified 78,192 post-menopausal women prescribed HRT and 1,604,822 not prescribed HRT. Post-propensity matching, each cohort comprised 67,874 patients. A total of 210 of the post-menopausal women prescribed HRT developed an ICD encounter diagnosis of gastroparesis at least 30 days after being prescribed HRT compared to post-menopausal women not prescribed HRT (OR = 1.23, 95% CI [1.01–1.51] *p*-value = 0.0395). These associations persisted in sensitivity analysis over 5 years (OR = 1.65, 95% CI [1.13–2.41] *p*-value = 0.0086). HRT was associated with increased GI symptoms, including early satiety (OR = 1.22, 95% CI [1.03–1.45] *p*-value = 0.0187), domperidone use (OR = 2.40, 95% CI [1.14–5.02] *p*-value = 0.0163), and undergoing gastric emptying studies (OR = 1.67, 95% CI [1.39–2.01] *p*-value < 0.0001). HRT is linked to an increased risk of developing an ICD encounter diagnosis of gastroparesis.

## 1. Introduction

Gastroparesis is a motility disorder of the stomach characterized as delayed gastric emptying in the absence of mechanical obstruction [1,2]. The symptoms of gastroparesis include nausea, vomiting, early satiety, postprandial fullness, bloating, and abdominal pain, which can significantly impact a patients’ quality of life and nutritional state [1,3]. Several etiologies can lead to the development of gastroparesis, including post-surgical, post-infectious, diabetic, idiopathic, autoimmune-disorder-related, neurological, and, in rare conditions, collagen-vascular-disorder-related [1,4].

A variety of mechanisms are thought to contribute to the pathophysiological development of gastroparesis, including impaired fundal accommodation, antral hypomotility, and insufficient pyloric relaxation [5]. Additionally, vagal and sympathetic nerve regulation and impairment to the enteric nervous system can lead to gastrointestinal dysregulation [4,6]. The proposed mechanisms that explain why this occurs include a loss of neuronal nitric oxide synthase (nNOS) expression, elevated oxidative stress [4,7,8], and loss of the interstitial cells of Cajal (ICC) [5].

The nitrergic system plays a critical role in regulating gastric motility, including gastric accommodation and pyloric relaxation. nNOS is expressed by inhibitory enteric neurons and is responsible for synthesizing nitric oxide (NO) [1]. nNOS is responsible for controlling the muscle tone of the lower esophagus, antrum, and pylorus and modulates the accommodative reflex of the fundus and the peristaltic reflex of the small intestine. The depletion of nNOS is thought to delay gastric emptying. A loss of NO is associated with loss of the ICC within the stomach. ICC are responsible for generating electrical slow waves in the stomach, enabling phasic contraction and peristalsis. Studies have demonstrated that a reduction in ICC leads to impaired electrical pace-making and delayed gastric emptying [1,4].

It has been recognized that women are more prone to developing gastroparesis, with a female to male ratio of 4:1. The prevalence of gastroparesis is 9.6 per 100,000 persons for men and 38 per 100,000 persons for women [9,10]. Yet, the underlying pathophysiology of why women are more susceptible to developing gastroparesis continues to remain incompletely elucidated.

Emerging evidence has demonstrated that sex hormones influence GI motility, as estrogen and progesterone receptors have been found within the GI tract [7,11]. Additionally, studies have shown that NO signaling is regulated by estrogen. It is thought that estrogen delays gastric emptying and decreases nNOS expression, leading to gastroparesis [12]. These steroid hormones fluctuate throughout the different stages of a woman’s life, including menstruation, pregnancy, and menopause. Pre-menopausal women are known to have higher levels of estrogen and progesterone than post-menopausal women. Studies have demonstrated that pre-menopausal women subsequently have slower gastric emptying than post-menopausal women [13,14]. After menopause, the levels of estrogen and progesterone decrease significantly, improving gastric emptying [13]. However, many post-menopausal women are prescribed HRT by their clinicians. Some studies have demonstrated that post-menopausal women taking HRT have delayed gastric emptying [7], whereas others have demonstrated HRT with estrogen to be a potential remedy for gastroparesis [15].

Given the conflicting evidence, the aim of our study was to investigate the association between HRT and developing an ICD encounter diagnosis of gastroparesis at least 30 days after post-menopausal women were prescribed HRT. Additionally, our study aimed to examine the effects of developing gastroparesis over time after HRT was initiated at 1, 3, and 5 years. Lastly, we investigated secondary endpoints, including the development of gastrointestinal symptoms, medication use, and diagnostic testing commonly seen in gastroparesis patients at least 30 days after post-menopausal women were prescribed HRT. 

## 2. Materials and Methods

### 2.1. Data Source

TriNetX is a platform that aggregates and de-identifies electronic healthcare record (EHR) data. For this study, we used the Global Collaborative Network in TriNetX, which brings together EHR data from across 106 healthcare organizations (HCOs) from 15 countries with over 120 million patients. This platform provides longitudinal patient information and allows for customizable cohort selection. It has built-in analytical tools which allow for patient-level analysis while maintaining patient privacy. Counts between 1 and 10 are reported as 10 for statistical de-identification in TriNetX [16]. The EHR information in TriNetX includes diagnoses, procedures, medications, laboratory values, and genomic information. TriNetX uses Systematized Nomenclature of Medicine Clinical Terms (SNOWMED-CT) coding for medical diagnosis and procedures. The International Classification of Disease, the Ninth and Tenth Revisions, Clinical Modification (ICD-9-CM and ICD-10-CM) are mapped into the SNOWMED-CT hierarchy for diagnosis. Current Procedural Terminology (CPT) codes are also used for procedures, Logical Observation Identifiers Name and Codes (LOINC) are used for laboratory tests, and prescription drugs are mapped onto RxNorm and Anatomical Therapeutic Chemical (ATC) codes. The data queries and analysis were conducted in the Global Collaborative Network on 9 September 2023.

### 2.2. Study Design

A retrospective cohort study was performed using the TriNetX platform, which included patients with at least some EHR-contributed information over a twenty-year span from 9 September 2003 to 9 September 2023. Post-menopausal women were included in this study, defined as women > 50 with at least one ICD encounter diagnosis of menopause. An age > 50 was chosen for the study group given the average age of menopause is 51 [17]. Patients of diverse racial and ethnic demographics were incorporated into this study. This included White, African American, Hispanic or Latina, Asian, American Indian or Alaska Native, and Native Hawaiian or Pacific Islander women. We divided our study group of post-menopausal women into two groups: post-menopausal women on HRT and post-menopausal women not on HRT. Hormone replacement therapy included the prescription drug classes of estrogen and progesterone. The index event was defined as the day of HRT initiation. The study outcomes included an ICD encounter diagnosis of gastroparesis at least 30 days after the index event. Secondary outcomes, including gastrointestinal symptoms, medications, diagnostic tests, and procedures, were examined 30 days after HRT initiation. These outcomes were identified based on diagnosis codes. Additionally, we performed a sensitivity analysis by analyzing the development of gastroparesis at 1, 3, and 5 years after the initiation of HRT.

### 2.3. Institutional Review Board

The Case Western Reserve University/MetroHealth Medical Center Institutional Review Board (IRB) designated the use of de-identified aggregated data from the TriNetX platform in the ways described in this manuscript as not involving human participants. The data from the TriNetX platform have also been deemed de-identified according to expert attestation, as defined in Section §164.514(a) of the HIPAA Privacy Rule [16]. The results of our study adhere to the guidelines outlined by the Strengthening the Reporting of Observational Studies in Epidemiology initiative for cohort studies [18].

### 2.4. Confounding Variables

To reduce the effect of confounding variables, we excluded patients with at least one ICD encounter diagnosis of functional dyspepsia and cyclic vomiting syndrome from our study. Additionally, we excluded patients with anatomical risk factors that are known to increase the risk of gastroparesis. Patients were excluded if they had any surgical procedure CPT code pertaining to the esophagus or stomach and bariatric surgery, Roux-en-Y gastrojejunostomy, gastric bypass, gastrectomy, or vagotomy procedures. One-to-one propensity matching was performed using the TriNetX built-in algorithm. We accounted for covariates including age at index event, gender, race, and ethnicity. Common risk factors that could contribute to gastroparesis, such as one or more ICD encounter diagnosis for diabetes, hemoglobin A1c (HgA1c), and BMI, were also accounted for during propensity matching.

### 2.5. Propensity Score Matching Details

The TriNetX platform employs input matrices of user-identified covariates and conducts logistic regression analysis to obtain propensity scores for individual subjects. Its 1:1 propensity score matching is based on the greedy nearest neighbor algorithm using a caliper width of 0.1 pooled SD. This randomization process allows investigators to eliminate bias and confounding factors when analyzing the desired data outcomes of interest. When propensity score matching is utilized, it involves assembling two groups of study participants, usually one group which received treatment, in our case HRT, and another which did not, while matching individuals with similar or identical propensity scores. The analysis of propensity-score-matched samples can approximate that of a randomized trial by directly comparing the outcomes between the two groups [19]. This study method has been validated in several studies [20,21,22,23,24].

### 2.6. Statistical Analysis

All demographic data present in this study from TriNetX were presented as either categorical data or continuous data with counts and percentages. Statistics were reported as means, standard deviations (SD), and proportions. One-to one propensity matching was performed on the covariates based on the greedy nearest neighbor algorithm utilizing a caliper width of 0.1 pooled SD on the TriNetX platform [25]. Statistical analysis was performed using TriNetX’s Measures of Association Analysis, which calculates and compares the fraction of patients with the selected outcome of interest. We obtained the number of patients with individual outcomes and the risks of the outcomes within the cohorts. Odds ratios (ORs) with a 95% confidence interval were obtained to compare cohorts. *p*-values < 0.05 were deemed statistically significant. The outcomes were generated at least 30 days after HRT was initiated. Patients were excluded from the analysis if their record included the outcome of interest prior to the beginning of the time window, in this case, 30 days. To enhance the validity of our data, we performed a sensitivity analysis by examining outcomes after 1:1 propensity score matching by analyzing the outcomes at 1, 3, and 5 years after the index event.

## 3. Results

**Figure 1:**
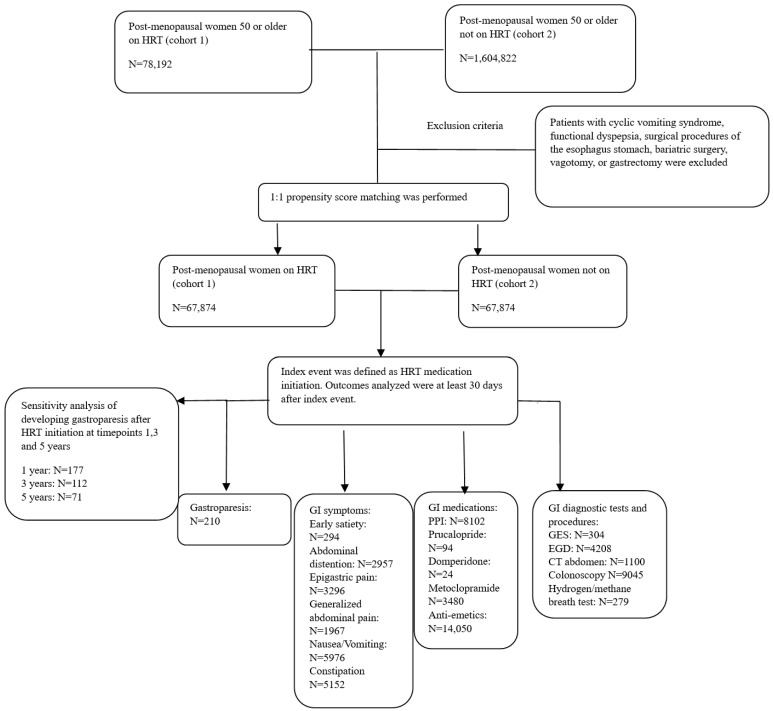
Flowchart diagram of study design.

A total of 78,192 patients were identified as post-menopausal women prescribed HRT (cohort 1), and 1,604,822 patients (cohort 2) were identified as post-menopausal women not prescribed HRT. After propensity matching and the exclusion criteria were applied, 67,874 patients were included in the study for each cohort. Additional patients were excluded from the results if they met the outcomes prior to the time window. The study paradigm, including the number of patients that developed each outcome, is demonstrated in Figure 1.

Their demographic features and comorbidities are summarized in Table 1. After propensity matching, the mean age of the post-menopausal women on HRT was 57.9 ± 6.7.

The most prominent race and ethnicity of the post-menopausal women prescribed HRT was Non-Hispanic White women. A total of 80.6% identified their race as White, and 75.9% identified their ethnicity as Non-Hispanic/Latina. The mean BMI and A1c of the post-menopausal women prescribed HRT were 26.8 ± 5.8 and 5.7 ± 1.1, respectively. The post-menopausal women not prescribed HRT had a higher mean BMI and A1c, of 28.9 ± 6.8 and 6.0 ± 1.4, respectively.

Two hundred and ten post-menopausal women developed at least one ICD encounter diagnosis of gastroparesis at least 30 days after being prescribed HRT compared to one hundred and seventy post-menopausal women not prescribed HRT. There was an increased odds of developing a diagnosis of gastroparesis (OR = 1.237, 95% CI [1.01–1.514] *p*-value = 0.0395) after taking HRT for 30 days. Additionally, there was an increased risk of 0.31% of developing a diagnosis of gastroparesis in the post-menopausal women prescribed HRT for 30 days. The sensitivity analysis evaluating the odds of developing an ICD encounter diagnosis of gastroparesis at 1, 3, and 5 years after being prescribed HRT in post-menopausal women is demonstrated in Table 2. These results revealed that the duration of HRT increases a woman’s odds of developing a diagnosis of gastroparesis over time.

### 3.1. Gastrointestinal Symptoms

The following outcomes, including early satiety, abdominal distention, epigastric pain, generalized abdominal pain, nausea and vomiting, constipation, and weight loss, were compared between post-menopausal women prescribed HRT and post-menopausal women not prescribed HRT. We found that post-menopausal women prescribed HRT for at least 30 days were associated with an increased statistically significant odds of developing a diagnosis of the gastrointestinal symptoms commonly associated with gastroparesis, as listed in Table 3. These included, most notably, an elevated odds of developing early satiety, abdominal distention, abdominal pain, nausea and vomiting, and constipation in comparison to the post-menopausal women not on HRT. Weight loss was not found to be statistically significant. 

### 3.2. Medications

We compared the prescriptions of various gastrointestinal (GI) medications that patients with a diagnosis of gastroparesis may be prescribed, as listed in Table 4. Our study found that the post-menopausal women prescribed HRT for at least 30 days were at an increased odds of being prescribed proton pump inhibitors (PPIs); pro-kinetic agents including prucalopride, domperidone, and metoclopramide; and antiemetics compared to the post-menopausal women not prescribed HRT.

### 3.3. Diagnostic Testing

Our study found that the post-menopausal women prescribed HRT for at least 30 days were at an increased odds of undergoing several gastrointestinal diagnostic tests and procedures commonly performed in patients with a diagnosis of gastroparesis in comparison to the post-menopausal women not prescribed HRT. The post-menopausal women prescribed HRT had an elevated risk of undergoing gastric emptying studies, esophagogastroduodenoscopy (EGD), computed tomography (CT) pf the abdomen, colonoscopy, and hydrogen/methane breath test, as listed in Table 5. Magnetic resonance imaging (MRI) of the abdomen was not found to be statistically significant. The observed increase in diagnostic test utilization, correlating with gastroparesis trends, suggests a potentially sensitive testing paradigm, mirroring the similar trends in gastroparesis manifestations within this cohort. 

## 4. Discussion

In summary, gastroparesis is a dysmotility disorder of the stomach that disproportionately affects women in comparison to men. The underlying pathophysiology as to why continues to remain a mystery [1,9]. Female sex hormones, including estrogen and progesterone, fluctuate throughout different hormonal stages in a woman’s lifetime, such as during menstruation, pregnancy, and menopause. It is thought that these sex hormones play a role in the underlying pathophysiology of the female predominance of gastroparesis [1,13,26].

Our study revealed that prescriptions for hormone replacement therapy were associated with an increased risk of developing an ICD encounter diagnosis of gastroparesis in post-menopausal women. The longer post-menopausal women had been prescribed HRT, the more their risk of developing gastroparesis increased. This association persisted despite controlling for confounding factors such as age, race, ethnicity, diabetes, BMI, and HgA1c. This is one of the largest database studies which examines the effect of hormone replacement therapy on the development of gastroparesis and investigated secondary endpoints such as the effects of HRT on GI symptoms, medications, diagnostic tests, and procedures. Our results support the results of other HRT-based studies which show that HRT supplementation increases the risk of developing gastroparesis [13].

### 4.1. Hormone Replacement Therapy and Racial/Ethnic Implications

Ethnic, racial, and gender differences among patients with a diagnosis of gastroparesis are important to consider, as symptomatology and etiology may differ between patients. The literature demonstrates that a higher proportion of Non-Hispanic Black people are diagnosed with diabetic gastroparesis in comparison to Non-Hispanic White people. Additionally, Non-Hispanic Black people were found to have more severe symptoms, including retching, vomiting, and a higher rate of hospitalizations. This study also demonstrated that a higher proportion of women have more severe symptoms than men [27].

The prior literature has also demonstrated that White women are more likely to be prescribed HRT in comparison to Black women [28]. These data are supported within the demographics of our study, listed in Table 1. Approximately 80.6% of White post-menopausal women were prescribed HRT after propensity matching in comparison to 5.6% of Black/African American post-menopausal women. In our study, 75.9% of post-menopausal women prescribed HRT identified as Non-Hispanic/Latino. Our study demonstrates the importance of taking into consideration the racial and ethnic backgrounds of patients who are prescribed HRT, as it may influence the etiology and symptomatology that gastroparesis patients may experience.

### 4.2. Hormone Replacement Therapy and GI Symptoms

The cardinal symptoms associated with gastroparesis include early satiety, postprandial fullness, bloating, abdominal pain, nausea, and vomiting [29]. The intensity of these GI symptoms can vary depending on the underlying etiology of gastroparesis. Another challenge is that the degree of gastric emptying in a gastric emptying scan (GES) often does not correlate with symptom severity [30]. Our study examined the association between post-menopausal women prescribed HRT and the development of these gastrointestinal symptoms commonly seen in patients with underlying gastroparesis. We found that the post-menopausal women prescribed HRT were statistically significant associated with an increased risk of the development of early satiety, abdominal distention, epigastric pain, generalized abdominal pain, nausea and vomiting, and constipation. These findings support the notion that HRT is associated with delayed gastric emptying, contributing to these GI symptoms.

### 4.3. Hormone Replacement Therapy and Medications

The treatment for gastroparesis ranges from conservative management with dietary modification, medications targeted at symptom management, and prokinetic agents to more invasive endoscopic or surgical interventions [30,31,32]. Common medications include antiemetic agents, which help decrease the symptoms of nausea and vomiting. These medications are commonly used in conjunction with prokinetic agents, which are the basis of treatment. This is because they accelerate gastric emptying by increasing contractility and improving gastropyloroduodenal motility [4,32]. Metoclopramide is a prokinetic and antinauseant agent that works as a potent central and peripheral dopamine receptor antagonist. Although it is approved by the FDA, it is only recommended for short-term treatment (4–12 weeks) due to its CNS-related side-effects, such as tardive dyskinesia, since it crosses the blood–brain barrier [33,34]. Another common agent used in the treatment of gastroparesis is domperidone, a dopamine (D2) antagonist. This medication enhances the contractility of the stomach; however, it only acts on the peripheral dopamine receptors [4,35]. Prucalopride is a serotonin 5-HT4 receptor agonist and is a potent gut motility stimulator, particularly of colonic motility, used in the treatment of constipation [4,36]. Our study found that post-menopausal women prescribed HRT were statistically significantly associated with an increased risk of being prescribed domperidone, metoclopramide, antiemetic agents, PPIs, and prucalopride. These data further support our hypothesis that HRT is associated with an increased risk of developing gastroparesis and may affect colonic dysmotility as well.

### 4.4. Hormone Replacement Therapy and Diagnostic Tests and Procedures

The definitive diagnostic test of choice for diagnosing gastroparesis is gastric emptying scintigraphy [1]. However, it takes a skilled clinician to recognize patients’ symptoms to arrive at the diagnosis of gastroparesis. In a recent study, the average time from symptom onset to a diagnosis of gastroparesis is approximately 5 years [37]. In our study, we found that post-menopausal women prescribed HRT were at an increased risk of having gastric emptying studies performed. We also found that post-menopausal women prescribed HRT were statistically significantly associated with an increased risk of undergoing diagnostic tests and procedures including EGD, CT of the abdomen, and colonoscopies.

A common complication seen in patients with gastroparesis is small intestinal bacterial overgrowth (SIBO) due to small intestinal hypomotility and impairment to the migratory motor complex (MCC) [38]. SIBO is often diagnosed using a hydrogen/methane breath test. This is because both hydrogen and methane are exclusively produced by microbial fermentation in the gut [39]. Our study found that post-menopausal women prescribed HRT were more likely to undergo diagnostic testing using a hydrogen/methane breath test. Thus, this raises the concern that HRT may not only be associated with delayed gastric emptying but also associated with small bowel dysmotility as well. Previous studies have demonstrated extragastric dysmotility, including small bowel and colonic transit delays, in patients with gastroparesis, which further supports our evidence [2].

### 4.5. Limitations

By conducting this study using aggregated EHR data, our study benefited from a large sample size, which reduced the risk of potential selection bias. Additionally, 1:1 propensity score matching allowed for randomization to control for confounding factors in our study. However, there are potential limitations to our study. Although we attempted to account for certain confounding factors that may influence the diagnosis of gastroparesis, it is possible that not all confounding factors may have been accounted for. Additionally, ICD encounter diagnosis codes are subject to the clinical decision of the providers; thus, the diagnostic tests performed to arrive at a diagnosis of gastroparesis remain unknown. Additionally, it is unknown whether patients prescribed HRT took their medication properly. However, we attempted to control for this somewhat by excluding patients with ICD encounter diagnoses codes for functional dyspepsia, cyclic vomiting syndrome, and surgical procedures on the esophagus and stomach to decrease misuse of the ICD encounter diagnosis code for gastroparesis.

## 5. Conclusions

In summary, our study demonstrated that HRT being prescribed in post-menopausal women is associated with an increased risk of developing an ICD encounter diagnosis of gastroparesis over time. Additionally, we found a significant association between HRT and elevated gastrointestinal side effects, medication use, and a higher likelihood of undergoing diagnostic tests and procedures compared to post-menopausal women not prescribed HRT. This study highlights the potential impact of estrogen and progesterone supplementation in HRT on gastrointestinal motility. Further investigation is warranted to elucidate the long-term effects of HRT on the gastrointestinal tract. Clinicians should be cognizant of the possible gastrointestinal complications when prescribing HRT to this population and take into consideration the ethnic and racial demographics of their patients.

## Figures and Tables

**Table 1:** Demographic characteristics of post-menopausal women on HRT (cohort 1) and post-menopausal women not on HRT (cohort 2).

Cohort 1 (N = 78,192) and Cohort 2 (N = 1,604,822) before Propensity Matching						Cohort 1 (N = 67,874) and Cohort 2 (N = 67,874) after Propensity Matching					
	Cohort	Mean ± SD	Patients	%Cohort	*p*-Value	SD	Mean ± SD	Patients	%Cohort	*p*-Value	SD
Age at Index	1	57.9 ± 6.7	67,874	100%	<0.001	0.735	57.9 ± 6.7	67,874	100%	0.992	<0.001
	2	63.7 ± 8.9	1,578,637	100%			57.9 ± 6.7	67,874	100%		
Female	1		67,874	100%	-	-		67,874	100%	-	-
	2		1,578,637	100%				67,874	100%		
White	1		54,710	80.6%	<0.001	0.202		54,710	80.6%	1	<0.001
	2		1,137,542	72.1%				54,710	80.6%		
Not Hispanic/Latino	1		51,502	75.9%	<0.001	0.189		51,502	75.9%	1	<0.001
	2		1,064,194	67.4%				51,502	75.9%		
Black or African American	1		3815	5.6%	<0.001	0.198		3815	5.6%	0.962	<0.001
	2		174,524	11.1%				3819	5.6%		
Hispanic or Latino	1		2860	4.2%	<0.001	0.141		2860	4.2%	0.925	0.001
	2		118,797	7.5%				2853	4.2%		
Asian	1		1626	2.4%	<0.001	0.082		1626	2.4%	1	<0.001
	2		60,399	3.8%				1626	2.4%		
American Indian/Alaska Native	1		141	0.2%	0.373	0.004		141	0.2%	0.764	0.002
	2		3540	0.2%				136	0.2%		
Native Hawaiian/Pacific Islander	1		79	0.1%	<0.001	0.052		79	0.1%	1	<0.001
	2		5892	0.4%				79	0.1%		
BMI	1	26.8 ± 5.8	32,415	47.8%	<0.001	0.402	26.8 ± 5.8	32,424	47.8%	0.987	<0.001
	2	29.3 ±6.8	395,212	25.0%			28.9 ± 6.8	32,421	47.8%		
Hgb A1c	1	5.7 ± 1.1	17,710	26.1%	<0.001	0.405	5.7 ± 1.1	17,711	26.1%	0.985	0.172
	2	6.3 ± 1.6	415,386	26.3%			6.0 ± 1.4	17,708	26.1%		
Diabetes Mellitus	1		5051	7.4%	<0.001	0.256		5051	7.4%	0.967	<0.001
	2		245,285	15.5%				5047	7.4%		

Abbreviations: Body mass index (BMI), hemoglobin A1c (Hgb A1c), standard deviation (SD), hormone replacement therapy (HRT).

**Table 2:** Sensitivity analysis analyzing the odds of developing gastroparesis in post-menopausal women at various timepoints after HRT initiation.

	OR	95% CI	*p*-Value
Year 1	1.44	1.144–1.814	0.0018
Year 3	1.602	1.188–2.159	0.0018
Year 5	1.653	1.132–2.414	0.0086

Abbreviations: confidence interval (CI), hormone replacement therapy (HRT), odds ratio (OR).

**Table 3:** Odds of post-menopausal women developing gastrointestinal symptoms after at least 30 days of HRT.

	OR	95% CI	*p*-Value	%RISK
Early satiety	1.227	[1.034–1.456]	0.0187	0.435%
Abdominal distention	1.441	[1.362–1.526]	<0.0001	4.59%
Epigastric pain	1.187	[1.127–1.249]	<0.0001	5.166%
Generalized abdominal pain	1.203	[1.126–1.285]	<0.0001	3.008%
Nausea and vomiting	1.138	[1.095–1.183]	<0.0001	9.849%
Constipation	1.219	[1.169–1.271]	<0.0001	8.484%
Weight loss	1.057	[0.986–1.132]	0.1180	2.564%

Abbreviations: odds ratio (OR), hormone replacement therapy (HRT), confidence interval (CI).

**Table 4:** Odds of post-menopausal women being prescribed gastrointestinal medications after at least 30 days of HRT.

	OR	95% CI	*p*-Value	%Risk
PPI	1.14	[1.102–1.179]	<0.0001	16.421%
Prucalopride	3.765	[2.422–5.853]	<0.0001	0.139%
Domperidone	2.402	[1.148–5.023]	0.0163	0.035%
Metoclopramide	1.112	[1.059–1.169]	<0.0001	5.487%
Antiemetics	1.21	[1.176–1.245]	<0.0001	29.704%

PPIs: rabeprazole, lansoprazole, esomeprazole, pantoprazole, omeprazole, dexlansoprazole. Antiemetics, including ondansetron, trimethobenzamide, prochlorperazine. Abbreviations: proton pump inhibitor (PPI), odds ratio (OR), confidence interval (CI), hormone replacement therapy (HRT).

**Table 5:** Odds of post-menopausal women undergoing diagnostic tests and procedures after at least 30 days of HRT.

	OR	95% CI	*p*-Value	%risk
GES	1.674	[1.393–2.013]	<0.0001	0.45%
EGD	1.199	[1.145–1.255]	<0.0001	6.624%
CT abdomen	1.25	[1.143–1.366]	<0.0001	1.678%
Colonoscopy	1.204	[1.166–1.244]	<0.0001	16.343%
Hydrogen/Methane breath test	1.783	[1.466–2.169]	<0.0001	0.413%
MRI abodomen	1.007	[0.931–1.09]	0.8580	1.875%

Abbreviations: Gastric emptying scan (GES), esophagogastroduodenoscopy (EGD), computed tomography (CT), magnetic resonance imaging (MRI), hormone replacement therapy (HRT), odds ratio (OR), confidence interval (CI).

## Data Availability

The data were collected from and analysis conducted on TriNetX, a global federated health research network providing access to de-identified electronic medical records from healthcare organizations. The data that support the findings of this study are available from the corresponding author upon reasonable request.
